# Estimated Influenza Illnesses and Hospitalizations Averted by Influenza Vaccination — United States, 2012–13 Influenza Season

**Published:** 2013-12-13

**Authors:** Joseph Bresee, Carrie Reed, Inkyu Kevin Kim, Lyn Finelli, Alicia Fry, Sandra S. Chaves, Erin Burns, Paul Gargiullo, Daniel Jernigan, Nancy Cox, James Singleton, Yusheng Zhai, Alissa O’Halloran, Katherine Kahn, Peng-Jun Lu, Tammy A. Santibanez

**Affiliations:** Influenza Div; Immunizations Svcs Div, National Center for Immunization and Respiratory Diseases, CDC

Influenza is associated with substantial morbidity and mortality each year in the United States. From 1976 to 2007, annual deaths from influenza ranged from approximately 3,300 to 49,000 (1). Vaccination against influenza has been recommended to prevent illness and related complications, and since 2010, the Advisory Committee on Immunization Practices has recommended that all persons aged ≥6 months be vaccinated against influenza each year (2). In 2013, CDC published a model to quantify the annual number of influenza-associated illnesses and hospitalizations averted by influenza vaccination during the 2006–11 influenza seasons (3). Using that model with 2012–13 influenza season vaccination coverage rates, influenza vaccine effectiveness, and influenza hospitalization rates, CDC estimated that vaccination resulted in 79,000 (17%) fewer hospitalizations during the 2012–13 influenza season than otherwise might have occurred. Based on estimates of the percentage of influenza illnesses that involve hospitalization or medical attention, vaccination also prevented approximately 6.6 million influenza illnesses and 3.2 million medically attended illnesses. Influenza vaccination during the 2012–13 season produced a substantial reduction in influenza-associated illness. However, fewer than half of persons aged ≥6 months were vaccinated. Higher vaccination rates would have resulted in prevention of a substantial number of additional cases and hospitalizations.

CDC used a model published in 2013 (3) to estimate an annual burden of influenza-associated outcomes prevented by influenza vaccination. All data inputs and estimates were stratified by age group: 6 months–4 years, 5–19 years, 20–64 years, and ≥65 years (Table 1). The disease burden during the 2012–13 influenza season was estimated in the following steps. First, laboratory-confirmed influenza-associated hospitalization rates were obtained from FluSurv-NET, a collaboration between CDC, the Emerging Infections Program Network, and selected health departments in 14 geographically distributed areas in the United States that conduct population-based surveillance. Hospitalization rates were adjusted for underreporting based on surveys conducted during the 2009 influenza pandemic; rates were increased by a factor of 2.74 for all age categories (3). Influenza illnesses were extrapolated from hospitalization data based on multipliers to reflect the percentage of ill persons who are hospitalized. Multipliers were age group–specific: 143.4 for 0–4 years; 364.7 for 5–19 years; 148.2 for 20–64 years, and 11.0 for ≥65 years. The percentage of ill persons who sought medical attention was estimated from the Behavioral Risk Factor Surveillance Survey (BRFSS) (3): 67% for persons aged 0–4 years; 51% for ages 5–19 years; 37% for ages 20–64 years, and 56% for ages ≥65 years. Second, 2012–13 vaccination coverage and vaccine effectiveness data were used to estimate the size of the susceptible population that was unprotected by vaccination and at risk of these outcomes. The rate of each outcome among susceptible persons was calculated as the number of estimated outcomes divided by the number of susceptible persons. This rate was then used to estimate the number of influenza-associated outcomes that would have occurred in a hypothetical, unvaccinated, susceptible population. Estimates of 2012–13 seasonal influenza vaccination coverage were based on self-report or parental report of vaccination status using data from the National Immunization Survey for children aged ≥6 months–17 years and BRFSS for adults aged ≥18 years,* and varied from 35.8 to 69.3, depending on the age group (Table 1). Vaccine effectiveness estimates were derived from the U.S. Influenza Vaccine Effectiveness (Flu VE) Network, a group of five academic institutions that conduct annual vaccine effectiveness studies, and varied from 32% among persons aged ≥65 years to 58% among children aged 6 months–4 years (4). Finally, the averted outcomes attributable to vaccination were estimated as the difference between outcomes in the hypothetical unvaccinated population and the observed population (3). The prevented fraction was calculated as the number of averted illnesses divided by the total illnesses that would have been expected in an unvaccinated population.

From October 2012 to May 2013, influenza vaccination resulted in an estimated 6.6 million (95% confidence interval [CI] = 4,011,725–10,551,756) fewer illnesses, 3.2 million (CI = 1,911,592–5,206,874) fewer medically attended illnesses, and 79,260 (CI = 39,530–136,744) fewer hospitalizations (Table 2). Overall, 17.3% (CI = 16.2%–18.0%) of adverse health outcomes associated with influenza were prevented. Although 29% of the averted illnesses and 39% of averted medically attended illnesses were among children aged 6 months–4 years and persons aged ≥65 years (two groups known to be at higher risk for complications), these two age groups accounted for 69% of averted hospitalizations. Vaccination had a substantial impact on averted hospitalizations in persons aged ≥65 years. Although persons aged ≥65 years accounted for 7% of the prevented illnesses and 8% of medically attended illnesses, 56% of all hospitalizations prevented were in those aged ≥65 years. If vaccination levels had reached the *Healthy People 2020* target of 70%, approximately 4.4 million illnesses, 1.8 million medically attended illnesses, and 30,000 additional hospitalizations might have been averted.

## Editorial Note

The 2012–13 influenza season was characterized as a moderately severe season, based on CDC influenza surveillance data.† During this season, rates of influenza-associated hospitalizations were 42.0 per 100,000 persons, compared with 7.7–23.4 per 100,000 during the previous three seasons. Among persons aged ≥65 years, hospitalization rates were three to seven times higher than rates observed for this age group during the previous three seasons. In addition, 169 influenza-associated pediatric deaths (deaths among persons aged <18 years) were reported to CDC, the highest number of reported deaths among this age group in a nonpandemic season since national reporting of influenza-associated pediatric deaths began in 2004. In this setting of a relatively high burden of severe disease, a 17% overall reduction in severe health outcomes resulted in a large number of prevented hospitalizations and medical visits for influenza that exceeded estimates of annual serious outcomes averted during influenza seasons from 2006 to 2011 (3). The fraction of outcomes averted was highest in children aged <5 years, among whom 30% of illness and hospitalizations were averted. The higher estimated prevented fraction in this group compared with older age groups was the result of higher vaccine coverage and vaccine effectiveness. Although vaccine effectiveness was lowest among persons aged ≥65 years, the relatively high vaccination coverage and high risk for severe outcomes resulted in substantial reductions in hospitalizations in this vulnerable group.

Fewer than half of persons in the United States are vaccinated each year (5), despite a recommendation for universal influenza vaccination for persons aged ≥6 months (3). Successful efforts to increase vaccination rates among all persons would increase the benefits of immunization programs on reducing illnesses. Strategies known to improve coverage should be encouraged. Those include ensuring that all those who visit a provider during the influenza season receive a vaccination recommendation and offer from their provider and using immunization information systems.§

Influenza vaccine effectiveness for the 2012–13 season was estimated to be 51% (CI = 45%–57%) (6), which is similar to estimates from recent seasons in the United States (2,4,6,7). Use of vaccines that are more effective would increase the number of averted illness and hospitalizations. However, despite a low measured vaccine effectiveness estimated to be 32% (CI = −5%–56%) among persons aged ≥65 years, vaccination likely produced substantial reductions in illness and hospitalizations because of the intensity of the 2012–13 epidemic and the relatively high vaccination coverage in this group compared with younger adults. Because most hospitalizations (8) and >90% of influenza deaths (1) occur in elderly persons, improving the ability of immunization programs to protect this vulnerable population will require vaccines with improved efficacy in elderly persons, along with continued efforts to increase vaccination rates. In 2010, a high-dose influenza vaccine (Fluzone HD, Sanofi Pasteur) was licensed for use in persons aged ≥65 years after prelicensure studies demonstrated superior immunogenicity compared with standard-dose vaccines (9). Final data from a recent efficacy trial are being analyzed.

The findings in this report are subject to at least six limitations. First, influenza vaccination coverage rates were derived from vaccination status reported by survey respondents, not vaccination records, and are subject to recall bias. Second, these rates are based on telephone surveys with relatively low response rates; therefore, selection bias might remain after weighting adjustments. Third, these surveys only cover the noninstitutionalized population. Fourth, estimates of the number of persons vaccinated based on these survey data exceeded the actual number of doses distributed, indicating coverage estimates used in this report overestimate averted illness resulting from vaccination (5). Fifth, the model only calculates outcomes directly averted by vaccination. If there is indirect protection from decreased exposure among unvaccinated persons in a partially vaccinated population (i.e., herd immunity), the model would underestimate the number of prevented illnesses. Also, although the impact of vaccination in preventing severe outcomes is most pronounced among persons aged ≥65 years, if vaccine effectiveness were lower among frail elderly persons, the model might have overestimated the effect in this group. Finally, adjustments for underreporting of influenza hospitalizations were based on studies conducted in 2009–10, as were the extrapolation of hospitalization rates to estimate rates of illness and medically attended illness. Because multipliers were calculated during a pandemic, if the ratio of hospitalizations to other outcomes or the underreporting of hospitalization rates were different in 2012–13 (e.g., through changes in health-seeking behaviors or testing practices), the model might have underestimated or overestimated the effect of vaccination.

What is already known on this topic?

Influenza vaccination has been a central tool for influenza prevention in the United States for >50 years. In 2012, CDC estimated that annual influenza vaccination resulted in 1.1–5.0 million fewer cases and 7,700–40,400 fewer hospitalizations annually during the 2005–11 influenza seasons.

What is added by this report?

Using surveillance data, vaccination coverage survey data and vaccine effectiveness estimates collected during the 2012–13 influenza season, estimates of the impact of influenza vaccination for the most recent season were generated. Vaccination during that season resulted in an estimated 79,260 fewer hospitalizations, 6.6 million fewer cases of influenza, and 3.2 million fewer medically attended cases.

What are the implications for public health practice?

The study supports the importance of influenza vaccination, but highlights the need for increasing coverage rates and more effective vaccines; efforts to increase vaccination rates can further reduce the burden of influenza.

Influenza vaccination prevents a substantial number of influenza-associated illnesses and hospitalizations. Although vaccines with increased effectiveness are needed, much can be done to maximize influenza prevention in the 2013–14 season.¶ In particular, efforts to increase vaccination rates will further reduce the burden of influenza. Although the timing and intensity of influenza circulation for the 2013–14 season cannot be predicted, peak weeks of influenza have occurred in January through March in >90% of seasons during the past 20 years, and significant circulation can occur as late as May. Therefore, vaccination should be offered now and as long as influenza continues to circulate.

## Figures and Tables

**TABLE 1:** Variables affecting impact of influenza vaccination, by age group — United States, 2012–13 influenza season

Age group (yrs)	Cumulative vaccine coverage (%)*		Vaccine effectiveness (%)†		Total population§	Cumulative hospitalization rate (per 100,000)¶	Estimated hospitalizations**		Estimated medically attended cases††		Estimated cases§§	
				
	%	(95% CI)	%	(95% CI)			No.	(95% CI)	No.	(95% CI)	No.	(95% CI)
6 mos–4	69.3	(67.8–70.8)	58.0	(40–71)	17,879,414	49.7	24,354	(15,224–38,206)	2,340,568	(1,466,829–3,701,433)	3,493,384	(2,183,709–5,480,282)
5–19	48.5	(47.6–49.4)	46.0	(32–57)	62,505,456	13.3	22,746	(14,172–35,852)	4,230,713	(2,612,263–6,656,229)	8,295,516	(5,168,531–13,075,561)
20–64	35.8	(35.2–36.4)	52.0	(43–60)	188,263,884	23.1	119,167	(78,995–177,656)	6,534,419	(4,292,193–9,776,843)	17,660,591	(11,707,096–26,328,640)
≥65	66.0	(65.2–66.8)	32.0	(0–56)	43,145,356	182.0	215,206	(142,909–316,950)	1,325,672	(871,647–1,968,414)	2,367,271	(1,572,003–3,486,454)
**All ages**	**44.7**	**(44.3–45.1)**	**51.0**	**(45–57)**	**311,794,110**	**42.0**	**381,474**	**(251,300–568,665)**	**14,431,371**	**(9,242,933–22,102,920)**	**31,816,763**	**(20,631,339–48,370,938)**

**Abbreviation:** CI = confidence interval.

* Season-cumulative vaccine overage rates calculated using data from the National Immunization Survey for children aged 6 months–17 years and from the Behavioral Risk Factor Surveillance System for adults aged ≥18 years. Model uses incremental monthly age-specific values. Estimates of the cumulative monthly proportion vaccinated through end of April of each season were developed using the Kaplan-Meier product limit method for receipt of most recent reported influenza vaccination. Negative lower 95% confidence intervals (CI) were revised to 0.

† Negative lower 95% CI intervals were revised to 0.

§ Calculated from U.S. Census Bureau annual estimates of the resident population by single year of age and sex for April 1, 2010 to July 1, 2012, available at http://factfinder2.census.gov/faces/tableservices/jsf/pages/productview.xhtml?pid=PEP_2012_PEPSYASEXN&prodType=table.

¶ Season-cumulative hospitalization rates calculated using data from the CDC Influenza Hospitalization Surveillance Network (FluSurv-NET). Model uses month-specific and age-specific values.

** Estimated using FluSurv-NET hospitalization rates adjusted for underreporting. The underreporting adjustment multiplier was calculated during the 2009–10 pandemic season and was 2.74 across age categories (3).

†† Based on the estimated number of cases and outpatient medically attended ratios by age group (3).

§§ Based on the estimated number of hospitalizations and age-specific case-hospitalization ratios for persons aged <65 years, and using a case-hospitalization ratio of 11:1 for persons aged ≥65 years (3).

**TABLE 2:** Estimated number of influenza cases averted by vaccination and the associated fraction prevented, by age group — United States, 2012–13 influenza season

Age group (yrs)	Averted hospitalizations		Averted, medically attended cases		Averted cases		Fraction prevented	
			
	No.	(95% CI)	No.	(95% CI)	No.	(95% CI)	%	(95% CI)
0–4	10,216	(5,994–16,502)	981,851	(575,222–1,591,166)	1,465,450	(859,735–2,367,044)	29.6	(28.0–30.2)
5–19	4,770	(2,869–7,722)	887,256	(529,333–1,437,481)	1,739,717	(1,046,532–2,816,363)	17.3	(16.8–17.8)
20–64	19,813	(12,887–30,107)	1,086,409	(698,241–1,666,804)	2,936,241	(1,909,887–4,461,808)	14.3	(14.0–14.5)
≥65	44,460	(17,779–82,413)	273,876	(108,797–511,422)	489,065	(195,570–906,541)	17.1	(10.5–21.3)
**All ages**	**79,260**	**(39,530–136,744)**	**3,229,393**	**(1,911,592–5,206,874)**	**6,630,473**	**(4,011,725–10,551,756)**	**17.3**	**(16.2–18.0)**

**Abbreviation:** CI = confidence interval.

## Footnotes

* Methods for estimating season-specific influenza vaccination coverage and descriptions of National Immunization Survey and BRFSS data are available at http://www.cdc.gov/mmwr/pdf/ss/ss6204.pdf.

† National, regional, and state influenza surveillance data can be viewed at http://www.cdc.gov/flu/fluactivitysurv.htm.

§ Evidence-based strategies for improving vaccination coverage are described in the *Community Guide for Preventive Services*, available at http://www.thecommunityguide.org/index.html.

¶ Full influenza immunization recommendations and a list of available vaccines are available at http://www.cdc.gov/flu/protect/vaccine/index.htm.

## References

[1] CDC (2010). Estimates of deaths associated with seasonal influenza—United States, 1976–2007. MMWR.

[2] CDC (2013). Prevention and control of seasonal influenza with vaccines. Recommendations of the Advisory Committee on Immunization Practices—United States, 2013–2014. MMWR.

[3] Kostova D, Reed C, Finelli L (2013). Influenza illness and hospitalizations averted by influenza vaccination in the United States, 2005–2011. PloS One.

[4] Treanor JJ, Talbot HK, Ohmit SE (2012). Effectiveness of seasonal influenza vaccines in the United States during a season with circulation of all three vaccine strains. Clin Infect Dis.

[5] CDC (2013). Flu vaccination coverage, United States, 2012–13 influenza season.

[6] CDC (2013). Advisory Committee on Immunization Practices (ACIP): summary report, June 19–20, 2013.

[7] Ohmit SE, Petrie JG, Malosh RE (2013). Influenza vaccine effectiveness in the community and the household. Clin Infect Dis.

[8] Zhou H, Thompson WW, Viboud CG (2012). Hospitalizations associated with influenza and respiratory syncytial virus in the United States, 1993–2008. Clin Infect Dis.

[9] Falsey AR, Treanor JJ, Tornieporth N, Capellan J, Gorse GJ (2009). Randomized, double-blind controlled phase 3 trial comparing the immunogenicity of high-dose and standard-dose influenza vaccine in adults 65 years of age and older. J Infect Dis.

